# Manganese deficiency or dietary manganese(III) oxide nanoparticle supplementation: consequences for hematology, and intestinal and brain immunity in rats

**DOI:** 10.3389/fimmu.2025.1528770

**Published:** 2025-04-08

**Authors:** Karolina Różaniecka-Zwolińska, Ewelina Cholewińska, Bartosz Fotschki, Jerzy Juśkiewicz, Katarzyna Ognik

**Affiliations:** ^1^ Department of Biochemistry and Toxicology, Faculty of Animal Sciences and Bioeconomy, University of Life Sciences in Lublin, Lublin, Poland; ^2^ Division of Food Science, Institute of Animal Reproduction and Food Research, Polish Academy of Sciences, Olsztyn, Poland

**Keywords:** manganese (III) oxide nanoparticles, manganese carbonate, immune status, inflammation, brain, jejunum

## Abstract

**Introduction:**

The study aimed to determine the effect of manganese (Mn) exclusion from the mineral mixture added to the rat diet and replacing the recommended level of MnCO_3_ (65 mg Mn/kg diet) with Mn_2_O_3_ nanoparticles (Mn_2_O_3_NPs) in the diet on blood hematology and selected immunological indices of the blood, jejunum, and brain.

**Methods:**

The experiment was conducted on twenty-four, Wistar rats divided into 3 equal groups. The control (K) group received a diet containing 65 mg/kg of additional Mn originating from the mineral mixture), group B (negative control) was fed a diet deprived of Mn from the mineral mixture, and group N was fed a diet containing 65 mg/kg Mn from Mn2O3NPs preparation. All rats received the experimental diets for 12 weeks. At the end of the experiment, samples of blood, jejunum, and brain were collected from all rats from each group.

**Results:**

Mn exclusion from the rat diet led to anemia, worsened the body’s immune response, and caused systemic and local inflammation as indicated e.g. by decreased RBC, HCT, and the level of HGB, and CRP in blood, CRP and IgA in the jejunum, and IgG in the brain as well as an increased level of IL-2, IgG and TNF-α in blood, and IL-6 in jejunum. In turn, replacing the recommended level of MnCO_3_ with Mn_2_O_3_NPs in the rat diet worsened the immune response and caused local inflammation in the brain as indicated by an increase in TNF-α level and Cp activity, as well as decreased levels of IgG. Analogical changes were not observed in the jejunum or systemic level.

**Discussion:**

The obtained results may suggest that the body has activated adaptive mechanisms that efficiently limit the spread of immune system disorders throughout the body.

## 1 Introduction

Manganese is one of the most important microelements, and its presence in the body determines its proper functioning. It acts as a cofactor of numerous enzymes involved in, among others, the process of gluconeogenesis (phosphoenolpyruvate decarboxylase and pyruvate carboxylase), antioxidant defense (manganese-dependent superoxide dismutase MnSOD), urea (arginase) and fatty acid (acetyl-CoA carboxylase) synthesis or ammonia metabolism in the brain (glutamine synthetase) (1, 2). Available literature also indicates that the presence of manganese is necessary for the proper synthesis of steroid hormones responsible especially for reproductive functions (3). It has also been proven that this element plays a key role in the processes of hematopoiesis and has a beneficial effect on the body’s immune functions. It has also been proven that this element plays a key role in the processes of hematopoiesis (4) and has anti-cancer effects, positively stimulating the body’s immune response by activating the cGAS-STING pathway, resulting in increased production of type I interferon (5). In addition, manganese is essential for the proper development, metabolism, integrity and regeneration of bone tissue, among others, by participating in the modulation of osteoblast and osteoclast activity, participating in the synthesis of collagen and proteoglycans as well as by participating in the complex regulation of signaling pathways and enzymatic reactions, it determines proper bone mineralization (6, 7). Therefore, it is very important to maintain the proper level of Mn in the body. In healthy people consuming a balanced diet, manganese deficiency is generally not observed because this element is commonly found in various food products such as fruits, vegetables, red meat, nuts, legumes, and whole grains (8, 9). On the other hand, Mn deficiency in the body was found in laboratory animals, whose diet is much less varied (1). Symptoms of manganese deficiency may vary somewhat depending on the species, but typically include impaired growth, skeletal abnormalities, depressed reproductive function, ataxia of the newborn, and faults in lipid and carbohydrate metabolism (10). There are also reports that manganese deficiency may worsen the body’s immunity by impairing the production of antibodies (11). Therefore, supplementation of this micronutrient is necessary in animals. Usually, it is done by including inorganic Mn salt, e.g. MnCO_3_, in a mineral mixture added to the diet. Unfortunately, the bioavailability of standard, inorganic Mn compounds introduced into the body through the digestive tract is relatively low because only about 1–5% of the consumed Mn is absorbed (12). Therefore, alternative forms of Mn that are more biocompatible and better utilized by the body are still being sought. Nanoparticles are therefore of increasing research interest. Our previous studies conducted on rats showed that Cu and Cr nanoparticles, due to their small size and specific physicochemical properties, penetrate the body more easily and are better utilized than their standard, inorganic counterparts (13–15). Therefore, it can be assumed that the use of Mn in the form of nanoparticles will have a favorable effect on the functioning of the body by regulating biological processes dependent on the attendance of this element.

Although, to the best of our knowledge, the mechanism of Mn interaction, especially in nanoparticle form, on immune status has not been clearly explained, its role in regulating immune response is undeniable. Huang et al. (16) report that manganese homeostasis plays a key regulatory role in innate immunity against infections, tumors, and acquired immunity. Mn^2+^ derived from the dissociation of inorganic salts as well as released from MnO_2_NPs enhances the activation of the cGAS-STING pathway to induce IRF3 phosphorylation, activate the NF-κB pathway, and promote IFN-I production to achieve an antiviral effect (17). There are also reports that Mn can sequester during infections and in this way, it can inhibit pathogen growth (18). It is also assumed that MnO_2_NPs may directly interact with the bacterial cell membrane or release Mn^2+^ ions, which participate in Fenton-type reactions generating large amounts of free radicals that damage the bacterial cell membrane. Consequently, the membrane becomes destabilized, leading to electrolyte/intracellular content leakage and reduced ATPase activity, ultimately resulting in bacterial death (16). Most of the studies conducted so far on manganese nanoparticles, however, focus primarily on determining their potential anticancer activity and their possible use in cancer diagnostics and therapy (19, 20). There is a lack of information regarding the impact of Mn nanoparticles on the immune status of healthy, uninfected, non-cancerous animals, especially when administered orally as a dietary supplement.

However, there is a certain risk associated with the use of nanoparticles as dietary supplements due to their potential toxicity. Although the available literature indicates that the mechanisms of Mn absorption in the intestines are strictly regulated and effectively protect the body against excessive Mn penetration from the gastrointestinal tract, and thus their toxicity (21), it cannot be ruled out that they will prove less effective or completely ineffective in the case of more bioavailable nanoparticles. This may be primarily due to the nature of nanoparticles, which are much smaller than inorganic Mn, so they can easily penetrate cell membranes and permeate cell organelles (22). As a result, an inappropriately selected dose of nanoparticles may result in their excessive accumulation in the body and the manifestation of their potential toxicity. Harischandra et al. (23) indicate that in the event of excessive exposure to Mn, its largest amounts accumulate in the central nervous system (especially in the basal ganglia of the brain) causing a disease called manganism, whose neurological symptoms are similar to those observed in Parkinson’s disease. It is assumed that Mn neurotoxicity results from disruption of neurotransmission, dopaminergic neurodegeneration, damage to cellular organelles, oxidative stress, and activation of cell death pathways (24, 25).

The study aimed to determine the effect of complete Mn exclusion from the mineral mixture added to the rat diet and replacing the recommended level of MnCO_3_ (65 mg Mn/kg diet) with Mn_2_O_3_NPs in the diet on blood hematology and selected immunological indices of the blood, jejunum, and brain.

## 2 Material and methods

### 2.1 Characterization of nanomaterial

Manganese (III) oxide nanoparticles (Mn_2_O_3_NPs) with a size of 40-60 nm were purchased from Sky Spring Nanomaterials Inc. (Houston, TX, USA).

### 2.2 Animal protocol and dietary treatments

All animal care and experimental protocols complied with the current laws governing animal experimentation in the Republic of Poland and by an ethical committee according to the European Convention for the Protection of Vertebrate Animals Used for Experimental and other Scientific Purposes, Directive 2010/63/EU for animal experiments (26), and were approved by the respective Local Institutional Animal Care and Use Committee (Approval No. 13/2022; Olsztyn, Poland, 16.03.2022). All efforts were made to minimize the suffering of the experimental animals.

Twenty-four, healthy, male, albino Wistar rats (Cmdb: WI) aged 4 weeks were divided into 3 groups (n=8, each). The number of animals in the group (n=8) was selected based on calculations determining the minimum group size necessary to obtain reliable research results that can be subjected to reliable statistical analysis. At the same time, this choice ensured compliance with the 3R principle (Replacement, Reduction, Refinement), guaranteeing an ethical approach to animal research. Rats were housed randomly and individually in stainless steel cages under a stable temperature (21-22°C), relative humidity of 60 ± 10%, a 12-h light-dark cycle, and a ventilation rate of 15 air changes per hour. For 12 weeks, the rats had free access to tap water and semi-purified diets, which were prepared and then stored at 4°C in hermetic containers until the end of the experiment (details in **Table 1**). The diets were modified from a casein diet for laboratory rodents recommended by the American Institute of Nutrition. Three experimental treatments were used to evaluate the effects of different treatments with supplemental Mn in the diet in the study. The control (K) group received a diet containing 65 mg/kg of additional Mn originating from the mineral mixture), group B (negative control) was fed a diet deprived of Mn from the mineral mixture, and group N was fed a diet containing 65 mg/kg Mn from the Mn_2_O_3_ nanoparticles (Mn_2_O_3_NPs) preparation (40-60 nm). Preparation of the experimental diets began with the calculation of the amount of standard MnCO_3_ and Mn_2_O_3_NPs that had to be added to the base diet to obtain diets containing 65 mg Mn/kg (this information is given in **Table 1**). In the case of the diet containing the standard form of Mn, the appropriately weighed MnCO_3_ was added directly to the mineral mixture, which was then mixed with the base diet. Because Mn_2_O_3_NPs occur as a fine, dusty powder that floats in the air, after weighing nanoparticles were not added directly to the mineral mixture but emulsified in the appropriate amount of rapeseed oil included in the diet. This was to avoid their loss and ensure consistency in manganese dosing, as well as to prepare a homogeneous diet because particles of such small size are difficult to distribute in a matrix containing larger particles. Mn_2_O_3_NPs emulsified with rapeseed oil were added to the basal diet previously mixed with the mineral mixture containing other essential microelements. The diet was then thoroughly mixed to obtain an appropriate dispersion of nanoparticles throughout the diet. The prepared experimental diets were re-mixed before administration to the rats to ensure the homogeneity of their components. The detailed composition of mineral mixtures used in all experimental groups has been provided in **Tables 2**, **3**.

**Table 1:** Composition of basal experimental diet fed to rats, %.

Ingredient	Content
*Unchangeable ingredients:*	
Casein^1^	14.8
DL-methionine	0.2
Cellulose^2^	8.0
Choline chloride	0.2
Rapeseed oil	8.0
Cholesterol	0.3
Vitamin mix^3^	1.0
Maize starch^4^	64.0
*Changeable ingredient:*	
Mineral mix (MX)^5^	3.5
*Calculated content:*	
Crude protein	13.5

^1^Casein preparation: crude protein 89.7%, crude fat 0.3%, ash 2.0%, and water 8.0%.

^2^α-Cellulose (SIGMA, Poznan, Poland), the main source of dietary fiber.

^3^AIN-93G-VM (47), g/kg mix: 3.0 nicotinic acid, 1.6 Ca pantothenate, 0.7 pyridoxine-HCl, 0.6 thiamin-HCl, 0.6 riboflavin, 0.2 folic acid, 0.02 biotin, 2.5 vitamin B-12 (cyanocobalamin, 0.1% in mannitol), 15.0 vitamin E (all-rac-α-tocopheryl acetate, 500 IU/g), 0.8 vitamin A (all-trans-retinyl palmitate, 500000 IU/g), 0.25 vitamin D-3 (cholecalciferol, 400000 IU/g), 0.075 vitamin K-1 (phylloquinone), 974.655 powdered sucrose.

^4^Maize starch preparation: crude protein 0.6%, crude fat 0.9%, ash 0.2%, total dietary fiber 0%, and water 8.8%.

^5^Changeable dietary ingredient to manganese level; mineral mixture (48) with standard Mn level and deprived of Mn, see **Tables 2**, **3**.

**Table 2:** Experimental schema *(provided manganese dosage was calculated taking into account MnCO_3_ in MX or Mn from Mn_2_O_3_ nanoparticles (Mn_2_O_3_NPs) preparation).

Group	12 weeks of feeding
B (Negative CONT, without Mn in MX)	A diet with MX deprived of Mn (n=8)
K (Control, with standard supplementation of Mn in MX)	A diet containing 65 mg/kg Mn from MnCO_3_ (n=8)
N (Nano-Mn, with standard supplementation of Mn but from novel nanoparticle source)	A diet containing 65 mg/kg Mn from Mn_2_O_3_ nanoparticles (n=8)

n=8, number of rats used in a particular feeding period.

*Experimental groups: B, during all twelve weeks of feeding the Mn deficient rats were given a diet with MX deprived of Mn (MnCO_3_ excluded from MX); K, the rats were fed a diet with standard mineral mixture (MX) resulting in 65 mg Mn (from MnCO_3_ in MX) per 1 kg of a diet during 12 weeks of feeding; N – the rats were given a diet containing 65 mg/kg Mn from Mn_2_O_3_NPs preparation per 1 kg of a diet during 12 weeks of feeding.

**Table 3:** Composition of mineral mixtures (MX) used in experimental diets.

	MX with standard Mn dosage^1^	MX deprived of Mn^2^
Calcium carbonate anhydrous CaCO_3_	357	357
Potassium phosphate monobasic K_2_HPO_4_	196	196
Potassium citrate C_6_H_5_K_3_O_7_	70.78	70.78
Sodium chloride NaCl	74	74
Potassium sulphate K_2_SO_4_	46.6	46.6
Magnesium oxide MgO	24	24
Microelements mixture^#^	18	18
Starch	To 1000g = 213.62	To 1000g = 213.62
^#^Microelements mixture:		
Ferric citrate [16,7% Fe]	31	31
Zinc carbonate ZnCO_3_ [56% Zn]	4.5	4.5
Manganous carbonate MnCO_3_ [44.4% Mn]	23.4	0
Copper carbonate CuCO_3_ [55.5% Cu]	1.85	1.85
Potassium iodate KJ	0.04	0.04
Citric acid C_6_H_8_O_7_	To 100 g = 39.21 g	To 100 g = 62.61

^1^given to the K group (12 weeks of feeding).

^2^given to the B and the N groups (12 weeks of feeding), but the N group was provided with the appropriate amount of Mn from Mn_2_O_3_ nanoparticles (Mn_2_O_3_NPs) preparation as an emulsion along with dietary rapeseed oil.

At the end of the experiment, the rats will be fasted for 12 hours and anesthetized *i.p*. with ketamine and xylazine (K, 100/kg BW; X, 10 mg/kg BW) according to recommendations for anesthesia and euthanasia of experimental animals. Following laparotomy, blood samples were collected from the caudal vena cava into heparinized tubes and EDTA tubes, and finally, the rats were euthanized by cervical dislocation. After that, the jejunum and brain were dissected. Although the ileum is the part of the small intestine with the most lymph nodes, which promotes the body’s immune defense, it was decided to use the jejunum for the study. The choice of jejunal tissue instead of ileal tissue for studies of immune status in conditions of insufficient Mn supply in the diet and the inclusion of Mn_2_O_3_NPs in the diet was dictated by the fact that the jejunum is a key site for the absorption of nutrients and minerals, which is of great importance in nutritional studies, especially on innovative dietary components, which undoubtedly are nanoparticles. Due to the potentially greater reactivity of Mn nanoparticles and its compounds and better bioavailability, it is the jejunal enterocytes that may be exposed to direct contact with the highest concentrations of Mn_2_O_3_NPs present in the food content, which in consequence may cause the most visible changes in the function and integrity of the intestines, which may be reflected in both the local and general immune status of the organism. In addition, important processes related to the immune response, such as antigen presentation and cytokine production, take place in the jejunum.

The brain was selected as the next tissue for the study because it is an organ for which Mn shows high affinity and significant toxicity. The blood plasma was prepared by solidification and low-speed centrifugation (350 × g, 10 min, 4°C). Samples of the collected organs were used to prepare tissue homogenates. For this purpose, 1 g of tissue from the appropriate organ was weighed and homogenized with 9 mL of PBS. Then the homogenate was centrifuged (3000 × g, 10 min, 4°C), and the supernatant was collected into a new tube. Plasma samples and tissue homogenates were stored at −80°C until analysis.

### 2.3 Blood and tissue analyses

Hematological examination was performed on whole blood using the ABACUS Jr VET Analyzer (DIATRON MI PLC, Budapest, Hungary), which included determination of the following parameters: total white blood cell (WBC) count, lymphocyte (LYM) count and percentage, medium-sized cell (MID) count and percentage, neutrophils (NEU) count and percentage, red blood cell count (RBC), hemoglobin (HGB), hematocrit (HCT), mean corpuscular volume (MCV), mean corpuscular hemoglobin (MCH), mean corpuscular hemoglobin concentration (MCHC), red cell distribution width (RDWc), platelet count (PLT), platelet percentage (PCT), mean platelet volume (MPV), and platelet distribution width (PDWc). Selected indicators of immune status were determined in blood plasma and homogenates of the jejunum and brain. In plasma, levels of interleukin-2 (IL-2; QY-E11521), interleukin-6 (IL-6; QY-E11509), tumor necrosis factor α (TNF-α; QY-E10880); immunoglobulins IgA (QY-E11193), IgM (QY-E11190) and IgG (QY-E11191), C-reactive protein (CRP; QY-E11591) and activity of ceruloplasmin (Cp; QY-E10365) were determined. In jejunum and brain homogenates, levels of interleukin-6 (IL-6; QY-E11509), tumor necrosis factor α (TNF-α; QY-E10880); immunoglobulins IgA (QY-E11193) and IgG (QY-E11191), C-reactive protein (CRP; QY-E11591), and activity of ceruloplasmin (Cp; QY-E10365) were determined. All immune parameters were determined using a commercial measurement enzyme-linked immunosorbent assay (ELISA) kit, following the protocol provided by the manufacturer (Shanghai Qayee Biotechnology Co., Ltd., Shanghai, China). Absorbances were measured at 450 nm via an ELISA reader (SunriseTM, Tecan Group Ltd., Männedorf, Switzerland).

### 2.4 Statistical analysis

Results were expressed as mean values with standard error of the mean (SEM). The statistical significance of differences between the experimental groups [Control (K), Nano-Mn (N), and Without Mn (B)] was assessed using a one-way analysis of variance (ANOVA) followed by a *post-hoc* test.

## 3 Results

### 3.1 The effect of excluding Mn from the diet (B group vs. K group)

Complete exclusion of Mn from the mineral mixture used in the rat diet resulted in decreased RBC, HGB, and HCT (P = 0.013, P = 0.025 and P = 0.019, respectively) in the blood (**Table 4**). This treatment also resulted in increased levels of IL-2, IgG, and TNF-α (P = 0.002, P < 0.001, and P = 0.032, respectively) and decreased levels of CRP (P < 0.001) and Cp activity (P = 0.016) in the blood plasma of rats (**Table 5**). In the jejunum tissue of rats fed a diet containing a mineral formula without added Mn, increased levels of IL-6 (P < 0.001) and Cp activity (P < 0.001) were noted, while CRP and IgA levels were decreased (P < 0.001 and P = 0.019, respectively) (**Table 6**). Complete exclusion of Mn from the mineral formula used in the rat diet also resulted in increased Cp activity (P < 0.001), while IgG levels (P < 0.001) were decreased in the brain (**Table 7**).

**Table 4:** Hematology parameters in rats fed experimental diets supplemented with manganese from MnCO_3_, Mn_2_O_3_-nanoparticles or without manganese supplementation^#^.

	Control diet (K)	Diet with Nano-Mn (N)	Diet without Mn (B)	Pooled SEM	P-value
WBC, 10^3^/µL	6.80 ± 0.392	6.23 ± 0.285	6.06 ± 398	0.211	0.183
LYM, 10^3^/µL	5.03 ± 0.405	4.53 ± 0.266	4.12 ± 0.202	0.184	0.057
MID, 10^3^/µL	0.146 ± 0.038	0.221 ± 0.078	0.250 ± 0.069	0.037	0.294
NEU, 10^3^/µL	1.63 ± 0.086	1.47 ± 0.068	1.69 ± 0.268	0.094	0.392
LYM, %	72.99 ± 2.38	72.40 ± 1.16	68.54 ± 2.18	1.169	0.148
MID, %	2.14 ± 0.493	3.75 ± 1.43	4.48 ± 1.34	0.675	0.194
NEU, %	24.86 ± 2.30	23.83 ± 0.986	27.01 ± 2.16	1.089	0.277
RBC, 10^6^/µL	9.60 ± 0.117^a^	9.30 ± 0.179^ab^	9.06 ± 0.094^b^	0.087	0.013
HGB, g/dL	14.03 ± 0.149^a^	13.30 ± 0.364^ab^	13.14 ± 0.173^b^	0.159	0.025
HCT, %	41.67 ± 0.474^a^	40.45 ± 0.785^ab^	39.55 ± 0.329^b^	0.359	0.019
MCV, fL	43.58 ± 0.175	43.50 ± 0.189	43.88 ± 0.125	0.097	0.143
MCH, pg	14.61 ± 0.159	14.26 ± 0.141	14.54 ± 0.143	0.087	0.127
MCHC, g/dL	33.66 ± 0.296	32.80 ± 0.307	33.21 ± 0.298	0.181	0.067
RDWc, %	19.75 ± 0.136^ab^	19.85 ± 0.105^a^	19.41 ± 0.114^b^	0.076	0.022
PLT, 10^3^/µL	686 ± 15.84	641 ± 39.25	602 ± 38.43	19.558	0.103
PCT, %	0.566 ± 0.017	0.553 ± 0.035	0.511 ± 0.031	0.017	0.213
MPV, fL	8.44 ± 0.038	8.66 ± 0.091	8.55 ± 0.113	0.051	0.095
PDWc, %	37.25 ± 0.224	37.46 ± 0.257	37.26 ± 0.427	0.175	0.658

^#^Group K fed a diet supplemented with 65 mg/kg Mn from MnCO_3_; Group N fed a diet supplemented with 65 mg/kg Mn from Mn_2_O_3_ nanoparticles; Group B fed a diet with a mineral mixture deprived of Mn.

^a,b^Mean values ± SEM (n=8 per group) within a row with unlike superscript letters are shown to be significantly different (P<0.05); Pooled SEM: pooled standard error of the mean (standard deviation for all rats divided by the square root of rat number, n=24).

WBC, total white blood cells; LYM, lymphocytes; MID, mid-sized cells; NEU, neutrophils; RBC, red blood cells; HGB, hemoglobin; HCT, hematocrit; MCV, mean corpuscular volume; MCH, mean corpuscular hemoglobin; MCHC, mean corpuscular hemoglobin concentration; RDWc, red cell distribution width; PLT, platelet count; PCT, platelet percentage; MPV, mean platelet volume; PDWc, platelet distribution width.

**Table 5:** Immune parameters in blood plasma of rats fed experimental diets supplemented with manganese from MnCO_3_, Mn_2_O_3_-nanoparticles or without manganese supplementation^#^.

	Control diet (K)	Diet with Nano-Mn (N)	Diet without Mn (B)	Pooled SEM	P-value
IL-2, ng/mL	297 ± 14.06^b^	266 ± 7.15^b^	347 ± 21.59^a^	10.984	0.002
CRP, ng/mL	97.82 ± 4.98^a^	60.00 ± 2.20^b^	67.77 ± 1.84^b^	3.863	<0.001
IgA, ng/mL	7117 ± 136.1	7719 ± 285.9	7752 ± 809.0	283.3	0.406
IgG, ng/mL	40014 ± 1041^b^	43148 ± 2450^b^	61777 ± 3721^a^	2477.1	<0.001
IgM, ng/mL	361 ± 21.93	299 ± 21.80	347 ± 22.17	13.325	0.072
IL-6, ng/mL	4.80 ± 0.183	5.03 ± 0.136	4.72 ± 0.100	0.084	0.167
TNF-α, ng/mL	131 ± 7.24^b^	141 ± 7.07^ab^	155 ± 6.61^a^	4.359	0.032
Cp, U/L	112 ± 4.85^a^	107 ± 4.76^ab^	93.51 ± 4.74^b^	3.107	0.016

^#^Group K fed a diet supplemented with 65 mg/kg Mn from MnCO_3_; Group N fed a diet supplemented with 65 mg/kg Mn from Mn_2_O_3_ nanoparticles; Group B fed a diet with a mineral mixture deprived of Mn.

^a,b^Mean values ± SEM (n=8 per group) within a row with unlike superscript letters are shown to be significantly different (P<0.05); Pooled SEM: pooled standard error of the mean (standard deviation for all rats divided by the square root of rat number, n=24).

IL-2, interleukin-2; CRP, C-reactive protein; IgA, immunoglobulin A; IgG, immunoglobulin G; IgM, immunoglobulin M; IL-6, interleukin-6; TNF-α, tumor necrosis factor α; Cp, ceruloplasmin.

**Table 6:** Immune parameters in the jejunum of rats fed experimental diets supplemented with manganese from MnCO_3_, Mn_2_O_3_-nanoparticles or without manganese supplementation^#^.

	Control diet (K)	Diet with Nano-Mn (N)	Diet without Mn (B)	Pooled SEM	P-value
CRP, ng/g	247 ± 11.08^a^	113 ± 10.48^c^	175 ± 4.31^b^	12.418	<0.001
IgA, ng/g	3237 ± 154.9^a^	2906 ± 183.3^ab^	2350 ± 331.8^b^	151.12	0.019
IgG, ng/g	4280 ± 156.0	4348 ± 189.3	4264 ± 108.1	85.724	0.721
IL-6, ng/g	0.032 ± 0.001^b^	0.050 ± 0.001^a^	0.047 ± 0.003^a^	0.002	<0.001
TNF-α, ng/g	0.462 ± 0.038^a^	0.249 ± 0.006^b^	0.501 ± 0.037^a^	0.029	<0.001
Cp, U/g	0.419 ± 0.016^b^	0.539 ± 0.084^b^	1.03 ± 0.092^a^	0.063	<0.001

^#^Group K fed a diet supplemented with 65 mg/kg Mn from MnCO_3_; Group N fed a diet supplemented with 65 mg/kg Mn from Mn_2_O_3_ nanoparticles; Group B fed a diet with a mineral mixture deprived of Mn.

^a,b^Mean values ± SEM (n=8 per group) within a row with unlike superscript letters are shown to be significantly different (P<0.05); Pooled SEM: pooled standard error of the mean (standard deviation for all rats divided by the square root of rat number, n=24).

CRP, C-reactive protein; IgA, immunoglobulin A; IgG, immunoglobulin G; IL-6, interleukin-6; TNF-α, tumor necrosis factor α; Cp, ceruloplasmin.

**Table 7:** Immune parameters in brains of rats fed experimental diets supplemented with manganese from MnCO_3_, Mn_2_O_3_-nanoparticles or without manganese supplementation^#^.

	Control diet (K)	Diet with Nano-Mn (N)	Diet without Mn (B)	Pooled SEM	P-value
CRP, ng/g	42.46 ± 3.92	47.82 ± 5.53	50.64 ± 5.20	2.815	0.280
IgA, ng/g	3087 ± 186.7	2734 ± 270.3	2585 ± 266.2	141.70	0.183
IgG, ng/g	15535 ± 1125^a^	7870 ± 214.9^c^	10991 ± 942.3^b^	808.65	<0.001
IL-6, ng/g	0.057 ± 0.002^ab^	0.061 ± 0.001^a^	0.053 ± 0.001^b^	0.001	0.003
TNF-α, ng/g	1.27 ± 0.026^b^	1.58 ± 0.035^a^	1.34 ± 0.075^b^	0.039	<0.001
Cp, U/g	0.934 ± 0.023^c^	1.65 ± 0.084^a^	1.13 ± 0.051^b^	0.070	<0.001

^#^ Group K fed a diet supplemented with 65 mg/kg Mn from MnCO_3_; Group N fed a diet supplemented with 65 mg/kg Mn from Mn_2_O_3_ nanoparticles; Group B fed a diet with a mineral mixture deprived of Mn.

^a,b,c^ Mean values ± SEM (n=8 per group) within a row with unlike superscript letters are shown to be significantly different (P<0.05); Pooled SEM: pooled standard error of the mean (standard deviation for all rats divided by the square root of rat number, n=24).

CRP, C-reactive protein; IgA, immunoglobulin A; IgG, immunoglobulin G; IL-6, interleukin-6; TNF-α, tumor necrosis factor α; Cp, ceruloplasmin.

### 3.2 The effect of replacing the recommended level of MnCO_3_ with Mn_2_O_3_ nanoparticles in the dietary mineral mixture (N group vs. K group)

Replacing the standard form of Mn (MnCO_3_) with Mn_2_O_3_NPs in the mineral mixture added to the rat diet resulted in a decrease in the level of CRP (P < 0.001) in the blood plasma (**Table 5**). Moreover, this treatment caused a reduction in the level of CRP and TNF-α (P < 0.001, both) and an increase in the level of IL-6 (P < 0.001) in the jejunum tissue of rats (**Table 6**). In the brain of rats receiving a diet containing Mn_2_O_3_NPs instead of the standard form of MnCO_3_, an increase in the level of TNF-α (P < 0.001) and Cp activity (P < 0.001) was noted, while the level of IgG (P < 0.001) was decreased (**Table 7**).

## 4 Discussion

An appropriate level of Mn in the body is necessary to maintain proper blood morphology (2, 27, 28). The results of the conducted studies are consistent with the above statements, as they showed that Mn deficiency in the diet caused by the complete exclusion of this micronutrient from the mineral mixture worsened hematological parameters by reducing the level of RBC, HGB, and HCT in the blood. Manganese is a cofactor of numerous enzymes directly or indirectly involved in the processes of erythropoiesis (porphobilinogen synthase, arginase, and carboxylase or glycosylases) (29), therefore a deficiency of this element in the diet could lead to a significant limitation of the synthesis of functional, catalytically active forms of these enzymes, as a result resulting in the occurrence of anemia symptoms.

Wu et al. (30) indicate that although manganese is necessary for maintaining proper immunity, its deficiency in the diet has a rather mild effect on the immune system, and its impact may include impaired production and secretion of antibodies. However, the results of the conducted studies indicate that the effect of manganese deficiency in the diet may have more serious immunological effects as it contributes to the development of systemic inflammation and increased activity of the immune system, as evidenced by the increased level of IgG, but also proinflammatory cytokines such as IL-2 and TNF-α in the blood plasma of the tested rats. The deterioration of the body’s systemic immunity due to insufficient Mn in the diet may result from the weakness of the immune functions and inflammation of individual organs under the influence of this procedure. The conducted studies showed the presence of local inflammation in the jejunal tissue of the tested rats, as evidenced by the increased level of the pro-inflammatory cytokine Il-6 in this tissue. Moreover, deficiency of manganese in the diet contributed to a deterioration of the immune response in both the small intestine and the brain, as evidenced by the corresponding decrease in the level of the main mucosal antibody IgA - in the intestinal tissue and IgG in the brain. Reduced levels of immunoglobulins and developing inflammation in the intestinal tissue may adversely affect the intestinal immune barrier, increasing susceptibility to infections and disorders of the intestinal microflora, which is consistent with the results of studies conducted by Jiang et al. (31). The reduced level of IgG in the brain may resulting from a weakening of IgG production by plasma cells or impaired transport of these antibodies across the blood-brain barrier due to Mn deficiency in the body. Inflammatory states are usually accompanied by increased production of acute phase proteins such as CRP or Cp. It is therefore interesting that in the case of the studied rats, a decrease in CRP levels was noted in the blood plasma and jejunal tissue, while in the case of Cp activity, its activity decreased in the blood plasma and increased in the tissue of the small intestine and the brain. The results suggest that the decreasing Cp activity should be considered in the context of its antioxidant effect. It is likely that in the conditions of MnSOD deficiency caused by Mn deficiency, the organism activates adaptive mechanisms as a result of which its antioxidant role was largely taken over by Cp. Increased activity of this protein in the brain and intestinal tissue may indicate the mobilization of the antioxidant response in conditions of increased ROS synthesis accompanying Mn deficiency. In turn, a decrease in its activity in the blood plasma may indicate that this protein is actively distributed to organs that currently show a high demand for antioxidant enzymes or that as a result of increased general oxidative stress, the reserves of active, catalytically functional protein are depleted at the systemic level. In addition, ROS produced in excess may directly or indirectly through the products of oxidation of cellular components activate numerous signaling pathways involved in the inflammatory response, such as NF-κB and MAPK (32, 33). As a result, there is an increased synthesis of proinflammatory cytokines that stimulate the development of inflammation (32). It has been proven that the secretion of CRP protein – one of the main acute phase proteins, occurs in the liver and is regulated mainly by IL-6, the action of which can be additionally enhanced by IL-1β and TNF-α (34). The results of our studies showed that despite the increase in IL-2 and TNF-α levels in the blood of rats, the IL-6 level remained at a level close to physiological. It can therefore be assumed that the observed reduction in CRP levels in the blood of the tested rats receiving a diet deficient in Mn resulted from an insufficient amount of IL-6 to induce increased synthesis of CRP protein despite the ongoing inflammation, e.g. due to the limitation of IL-6 mRNA stability caused by excessive amounts of ROS. It is also possible that as a result of probable oxidative stress, hepatocyte damage occurred, which reduced their ability to produce CRP protein. It is worth emphasizing also that the main organ responsible for the production of CRP protein is the liver, not the jejunum. The observed increase in IL-6 levels in intestinal tissue, which probably occurred as a result of activation of the NF-κB pathway in intestinal epithelial cells and macrophages under the influence of increased ROS synthesis, may not have been sufficient to induce a systemic increase in CRP, especially if additional limitations in IL-6 transport to the liver were also present.

Although manganese is necessary for the proper functioning of the body, it can have potentially toxic effects, especially when present in excess (4). Therefore, supplementation of microelements in the form of nanoparticles is particularly risky, even when the recommendations regarding their dosage are followed, as there are some reports that they can easily cross standard barriers in the body, including the blood-brain barrier (13, 14). However, this study did not show that replacing standard MnCO_3_ with Mn_2_O_3_NPs in the rat diet harmed the systemic immune response confirmed by the lack of negative change of immunological parameters level in the rats’ blood. Moreover, this procedure favorably reduced the level of CRP in the blood plasma, which may indicate a systemic effect of the nanoparticles, which affect immune homeostasis by modifying signaling pathways.

However, this study also demonstrated that Mn_2_O_3_NPs inclusion in the rat diet may cause adverse local inflammatory changes in individual organs. The results have shown that the brain is an organ particularly sensitive to the immunotoxic effects of manganese nanoparticles as evidenced by increased levels of proinflammatory TNF-α and the activity of Cp as an acute-phase protein but also an unfavorable decrease in brain IgG levels. It is consistent with the results of studies by other authors (35–38). Meng et al. (35) found that intratracheal injection of MnO_2_NP in rats caused dose-dependent degradation of hippocampal and choroid plexus (CP) cell structure and impaired learning and memory ability. According to the results of the study by Afeseh Ngwa et al. (36), manganese nanoparticles show high affinity for N27 dopaminergic neurons, where they induce a time-dependent upregulation of the transport protein-transferrin. Sun et al. (37) in *in vitro* studies demonstrated that MnO_2_ nanoparticles can induce inflammatory changes in BV2 microglial cells by increasing the level of intracellular ROS and further activation of the p38 MAPK pathway. Afeseh Ngwa et al. (36) indicate that the mechanism of nanoparticle neurotoxicity also includes autophagy and activation of the apoptosis signaling pathway, as evidenced by the proteolytic cleavage of proapoptotic protein kinase Cδ (PKCδ) dependent on caspase, as well as phosphorylation of the activation loop (36). It is consistent with the results of Sadeghi et al. (39) who administered MnO_2_-NPs (30-60 nm) to rats for 15 days and observed a dose-dependent increase in oxidative stress, a decrease in catecholamine levels, and an increase in the number of apoptotic and necrotic cells in the hippocampal tissue, as well as behavioral changes similar to depression in the animals studied. Mehdizadeh et al. (38) in an *in vitro* study conducted on SH-SY5Y cells also demonstrated that exposure to manganese nanoparticles (1–200 μg/ml Mn for 24 h) activates caspase-3 and caspase-9, increases the Bax/Bcl-2 ratio, and leads to the adoption of a more compact structure by the microtubule-stabilizing protein tau in neurons, which indicates the activation of apoptotic pathways. Meng et al. (35) also indicate that adverse changes, including inflammatory changes, in brain tissue may be related to transcriptome disturbances caused by MnO_2_-NP, including primary changes in the expression of transporters, ion channel proteins, and ribosomal proteins. Higher neurotoxicity of nanoparticles of Mn or its chemical compounds may probably be due to their high bioavailability, because after absorption from the gastrointestinal tract are rapidly transported to high-energy organs containing numerous mitochondria - in particular to the liver, pancreas, and especially the brain, where they quickly gain of high levels (40). Our study also showed that replacing MnCO_3_ with Mn_2_O_3_NPs in the rat diet increased the level of pro-inflammatory cytokine IL-6 in the jejunum tissue of the tested rats. This could suggest that Mn_2_O_3_NPs included in the diet have an immunotoxic effect not only on the brain but also on the jejunum. Studies conducted by Cheng et al. (41–43) indicate that xenobiotics introduced into the body orally can affect intestinal cells, causing damage and inducing inflammation through various mechanisms. These include increased synthesis of reactive oxygen species (ROS) leading to disruption of autophagic flux and activation of the apoptosis pathway (41, 42) and disruption of the intestinal microbial balance, which can lead to abnormal activation of AhR and NF-κB, resulting in intestinal inflammation (43). Saod et al. (44) showed that MnONP has potent antibacterial activity against pathogenic bacteria such as *Escherichia coli*, *Klebsiella pneumoniae*, and *Pseudomonas aeruginosa*, which may be due to their tendency to generate free radicals promoting damage to bacterial cell membranes. However, the presence of manganese nanoparticles in food may have a biocidal effect not only on pathogenic bacteria, but also on beneficial bacteria, leading to an unfavorable reconstruction of the intestinal microflora and the occurrence of inflammation, which probably occurred in the case of the rats we studied. Interestingly, however, in the intestinal tissue of rats receiving a diet containing Mn_2_O_3_NPs instead of MnCO_3_, a simultaneous decrease in CRP and TNF-α levels was found, which rather excludes the presence of inflammation and even suggests an enhancement of local defense mechanisms in response to the presence of Mn_2_O_3_NPs. The difference in the response of the brain and jejunum to Mn_2_O_3_NPs may result from different mechanisms of transport and accumulation of nanoparticles in different tissues and differences in tissue microenvironments. The small intestine, being the first point of contact with the diet, may better cope with the attendance of Mn_2_O_3_NPs than the brain through defense mechanisms, such as mucus secretion or increased enzymatic activity. In light of the presented results, it can therefore be assumed that under the influence of the inflammation initiated by Mn_2_O_3_NPs in the brain, adaptive mechanisms that efficiently prevented the spread of these changes at the systemic level were activated.

Because the results of our studies and the results of other authors (35, 36, 38, 39) indicate that nanoparticles of manganese and its compounds exhibit neurotoxic effects, their use as a dietary supplement does not seem to be safe. Moreover, the detailed bibliometric analyses conducted by Ghassemi Nejad et al. (45) showed that currently there are also other alternative forms of manganese, such as organic chelates. Similarly to nanoparticles, they are characterized by high bioavailability, but at the same time, they exhibit lower toxicity, because they are less susceptible to excessive accumulation, which reduces the risk of oxidative stress. Khoshbin et al. (46) showed that the inclusion of manganese-methionine chelate (Mn-Met) in the diet of laying hens increased the bioavailability of this element and enhanced antioxidant protection and immune response, and these benefits were greater when Mn-Met was added to the diet in higher proportions (50 or 75% of the recommended dietary amount of Mn). Further comparative studies are therefore necessary to determine the mechanisms behind the differential effects of nanoparticles and manganese chelates on antioxidant pathways and immune response. Their results may pave the way for hybrid manganese preparations that combine nanoparticles’ rapid action with chelates’ safety. In summary Mn, exclusion from the rat diet led to anemia, disrupted the body’s immune response, and caused systemic and local inflammation including the jejunum and brain. It was therefore confirmed that maintaining proper Mn homeostasis in the body is necessary to ensure the proper functioning of the immune system. In turn, replacing the recommended level of MnCO_3_ with Mn_2_O_3_NPs in the rat diet disrupted the immune response and caused local inflammation in the brain, which was not observed at the jejunum or systemic level. The obtained results may suggest that the body has activated adaptive mechanisms that efficiently limit the spread of immune system disorders throughout the body. Therefore, one should be very careful when introducing Mn_2_O_3_NPs supplementation into the rat diet.

## Data Availability

The raw data supporting the conclusions of this article will be made available by the authors, without undue reservation.

## References

[1] Erikson KM Aschner M. Manganese: its role in disease and health. Met Ions Life Sci. (2019), 19:/books/9783110527872/9783110527872–016/9783110527872-016.xml. doi: 10.1515/9783110527872-016 30855111

[2] Li L Yang X. The essential element manganese, oxidative stress, and metabolic diseases: links and interactions. Oxid Med Cell Longev. (2018) 2018:7580707. doi: 10.1155/2018/7580707 29849912 PMC5907490

[3] Studer JM Schweer WP Gabler NK Ross JW. Functions of manganese in reproduction. Anim Reprod Sci. (2022) 238:106924. doi: 10.1016/j.anireprosci.2022.106924 35121412

[4] Oliveira DC Nogueira-Pedro A Santos EW Hastreiter A Silva GB Borelli P. A review of select minerals influencing the haematopoietic process. Nutr Res Rev. (2018) 31:267–80. doi: 10.1017/S0954422418000112 29983125

[5] Han X Leng C Zhao S Wang S Chen S Wang S. Development and verification of a manganese metabolism- and immune-related genes signature for prediction of prognosis and immune landscape in gastric cancer. Front Immunol. (2024) 15:1377472. doi: 10.3389/fimmu.2024.1377472 38807601 PMC11131102

[6] Rondanelli M Faliva MA Peroni G Infantino V Gasparri C Iannello G. Essentiality of manganese for bone health: an overview and update. Nat Prod Commun. (2021) 16:1–8. doi: 10.1177/1934578X211016649

[7] Taskozhina G Batyrova G Umarova G Issanguzhina Z Kereyeva N. The manganese-bone connection: investigating the role of manganese in bone health. J Clin Med. (2024) 13:4679. doi: 10.3390/jcm13164679 39200820 PMC11355939

[8] Ross AC Caballero B Cousins RJ Tucker KL Ziegler TR. Modern nutrition in health and disease: Eleventh edition. Waltham, Massachusetts: Wolters Kluwer Health Adis (ESP (2012).

[9] Chen P Bornhorst J Aschner M. Manganese metabolism in humans. Front Biosci (Landmark Ed). (2018) 23:1655–79. doi: 10.2741/4665 29293455

[10] EFSA. Scientific Opinion on the safety and efficacy of manganese compounds (E5) as feed additives for all animal species: manganous oxide, based on a dossier submitted by Poortershaven Industriële Mineralen BV. EFSA Journal. (2013) 11(8):3325. doi: 10.2903/j.efsa.2013.3325

[11] Haase H. Innate immune cells speak manganese. Immunity. (2018) 48:616–8. doi: 10.1016/j.immuni.2018.03.031 29669242

[12] Aschner JL Aschner M. Nutritional aspects of manganese homeostasis. Mol Aspects Med. (2005) 26:353–62. doi: 10.1016/j.mam.2005.07.003 PMC6309959 16099026

[13] Cholewińska E Ognik K Fotschki B Zduńczyk Z Juśkiewicz J. Comparison of the effect of dietary copper nanoparticles and one copper (II) salt on the copper biodistribution and gastrointestinal and hepatic morphology and function in a rat model. PloS One. (2018) 13:e0197083. doi: 10.1371/journal.pone.0197083 29758074 PMC5951546

[14] Marzec A Cholewińska E Fotschki B Juśkiewicz J Stępniowska A Ognik K. Are the biodistribution and metabolic effects of copper nanoparticles dependent on differences in the physiological functions of dietary fibre? Ann Anim Sci. (2024) 25(1):175–87. doi: 10.2478/aoas-2024-0057

[15] Stępniowska A Tutaj K Juśkiewicz J Ognik K. Effect of a high-fat diet and chromium on hormones level and Cr retention in rats. J Endocrinol Invest. (2022) 45:527–35. doi: 10.1007/s40618-021-01677-3 PMC8850218 34550535

[16] Huang Y Ruan Y Ma Y Chen D Zhang T Fan S. Immunomodulatory activity of manganese dioxide nanoparticles: Promising for novel vaccines and immunotherapeutics. Front Immunol. (2023) 14:1128840. doi: 10.3389/fimmu.2023.1128840 36926351 PMC10011163

[17] Wang C Guan Y Lv M Zhang R Guo Z Wei X. Manganese Increases the Sensitivity of the cGAS-STING Pathway for Double-Stranded DNA and Is Required for the Host Defense against DNA Viruses. Immunity. (2018) 48:675–87. doi: 10.1016/j.immuni.2018.03.017 29653696

[18] Kehl-Fie TE Skaar EP. Nutritional immunity beyond iron: a role for manganese and zinc. Curr Opin Chem Biol. (2010) 14:218–24. doi: 10.1016/j.cbpa.2009.11.008 PMC2847644 20015678

[19] Zhu C Ma Q Gong L Di S Gong J Wang Y. Manganese-based multifunctional nanoplatform for dual-modal imaging and synergistic therapy of breast cancer. Acta Biomater. (2022) 141:429–39. doi: 10.1016/j.actbio.2022.01.019 35038584

[20] Pan S Sun Z Zhao B Miao L Zhou Q Chen T. Therapeutic application of manganese-based nanosystems in cancer radiotherapy. Biomaterials. (2023) 302:122321. doi: 10.1016/j.biomaterials.2023.122321 37722183

[21] Roth JA. Homeostatic and toxic mechanisms regulating manganese uptake, retention, and elimination. Biol Res. (2006) 39:45–57. doi: 10.4067/s0716-97602006000100006 16629164

[22] Kumar N Thorat ST Reddy KS. Multi biomarker approach to assess manganese and manganese nanoparticles toxicity in *Pangasianodon hypophthalmus* . Sci Rep. (2023) 13:8505. doi: 10.1038/s41598-023-35787-0 37231182 PMC10213040

[23] Harischandra DS Ghaisas S Zenitsky G Jin H Kanthasamy A Anantharam V. Manganese-induced neurotoxicity: new insights into the triad of protein misfolding, mitochondrial impairment, and neuroinflammation. Front Neurosci. (2019) 13:654. doi: 10.3389/fnins.2019.00654 31293375 PMC6606738

[24] Balachandran RC Mukhopadhyay S McBride D Veevers J Harrison FE Aschner M. Brain manganese and the balance between essential roles and neurotoxicity. J Biol Chem. (2020) 295:6312–29. doi: 10.1074/jbc.REV119.009453 PMC7212623 32188696

[25] Milatovic D Yin Z Gupta RC Sidoryk M Albrecht J Aschner JL. Manganese induces oxidative impairment in cultured rat astrocytes. Toxicol Sci. (2007) 98:198–205. doi: 10.1093/toxsci/kfm095 17468184

[26] Directive 2010/63/EU of the European Parliament and of the Council of 22 September 2010 on the protection of animals used for scientific purposes. OJEU. (2010) L276:20.10.2010:33–79.

[27] Katz N Rader DJ. Manganese homeostasis: from rare single-gene disorders to complex phenotypes and diseases. J Clin Invest. (2019) 129:5082–5. doi: 10.1172/JCI133120 PMC6877306 31682237

[28] Liu M Sun X Chen B Dai R Xi Z Xu H. Insights into manganese superoxide dismutase and human diseases. Int J Mol Sci. (2022) 23:15893. doi: 10.3390/ijms232415893 36555531 PMC9786916

[29] Avila DS Puntel RL Aschner M. Manganese in health and disease. Met Ions Life Sci. (2013) 13:199–227. doi: 10.1007/978-94-007-7500-87 24470093 PMC6589086

[30] Wu Q Mu Q Xia Z Min J Wang F. Manganese homeostasis at the host-pathogen interface and in the host immune system. Semin Cell Dev Biol. (2021) 115:45–53. doi: 10.1016/j.semcdb.2020.12.006 33419608

[31] Jiang WD Tang RJ Liu Y Kuang SY Jiang J Wu P. Manganese deficiency or excess caused the depression of intestinal immunity, induction of inflammation and dysfunction of the intestinal physical barrier, as regulated by NF-κB, TOR and Nrf2 signalling, in grass carp (Ctenopharyngodon idella). Fish Shellfish Immunol. (2015) 46:406–16. doi: 10.1016/j.fsi.2015.06.007 26072140

[32] Forrester SJ Kikuchi DS Hernandes MS Xu Q Griendling KK. Reactive oxygen species in metabolic and inflammatory signaling. Circ Res. (2018) 122:877–902. doi: 10.1161/CIRCRESAHA.117.311401 29700084 PMC5926825

[33] Mittal M Siddiqui MR Tran K Reddy SP Malik AB. Reactive oxygen species in inflammation and tissue injury. Antioxid Redox Signal. (2014) 20:1126–67. doi: 10.1089/ars.2012.5149 PMC3929010 23991888

[34] Ngwa DN Pathak A Agrawal A. IL-6 regulates induction of C-reactive protein gene expression by activating STAT3 isoforms. Mol Immunol. (2022) 146:50–6. doi: 10.1016/j.molimm.2022.04.003 PMC9811655 35430542

[35] Meng CY Ma XY Xu MY Pei SF Liu Y Hao ZL. Transcriptomics-based investigation of manganese dioxide nanoparticle toxicity in rats’ choroid plexus. Sci Rep. (2023) 13:8510. doi: 10.1038/s41598-023-35341-y 37231062 PMC10213021

[36] Afeseh Ngwa H Kanthasamy A Gu Y Fang N Anantharam V Kanthasamy AG. Manganese nanoparticle activates mitochondrial dependent apoptotic signaling and autophagy in dopaminergic neuronal cells. Toxicol Appl Pharmacol. (2011) 256:227–40. doi: 10.1016/j.taap.2011.07.018 PMC3205240 21856324

[37] Sun X Qin X Liang G Chang X Zhu H Zhang J. Manganese dioxide nanoparticles provoke inflammatory damage in BV2 microglial cells via increasing reactive oxygen species to activate the p38 MAPK pathway. Toxicol Ind Health. (2024) 40:244–53. doi: 10.1177/07482337241242508 38518383

[38] Mehdizadeh P Fesharaki SSH Nouri M Ale-Ebrahim M Akhtari K Shahpasand K. Tau folding and cytotoxicity of neuroblastoma cells in the presence of manganese oxide nanoparticles: Biophysical, molecular dynamics, cellular, and molecular studies. Int J Biol Macromol. (2019) 125:674–82. doi: 10.1016/j.ijbiomac.2018.11.191 30468808

[39] Sadeghi L Babadi VY Tanwir F. Manganese dioxide nanoparticle induces Parkinson like neurobehavioral abnormalities in rats. Bratisl Lek Listy. (2018) 119:379–84. doi: 10.4149/BLL_2018_070 29947239

[40] Singh SP Kumari M Kumari SI Rahman MF Mahboob M Grover P. Toxicity assessment of manganese oxide micro and nanoparticles in Wistar rats after 28 days of repeated oral exposure. J Appl Toxicol. (2013) 33:1165–79. doi: 10.1002/jat.2887 23702825

[41] Cheng X Liang J Wu D Guo X Cao H Zhang C. Blunting ROS/TRPML1 pathway protects AFB1-induced porcine intestinal epithelial cells apoptosis by restoring impaired autophagic flux. Ecotoxicol Environ Saf. (2023) 257:114942. doi: 10.1016/j.ecoenv.2023.114942 37086622

[42] Cheng X Hu Y Kuang J Guo X Cao H Wu H. Berberine alleviates high-energy and low-protein diet-induced fatty liver hemorrhagic syndrome in laying hens: insights from microbiome and metabolomics. Poult Sci. (2024) 103:103968. doi: 10.1016/j.psj.2024.103968 38959643 PMC11269790

[43] Cheng X Chen J Guo X Cao H Zhang C Hu G. Disrupting the gut microbiota/metabolites axis by Di-(2-ethylhexyl) phthalate drives intestinal inflammation via AhR/NF-κB pathway in mice. Environ pollut. (2024) 343:123232. doi: 10.1016/j.envpol.2023.123232 38171427

[44] Saod WM Hamid LL Alaallah NJ Ramizy A. Biosynthesis and antibacterial activity of manganese oxide nanoparticles prepared by green tea extract. Biotechnol Rep (Amst). (2022) 34:e00729. doi: 10.1016/j.btre.2022.e00729 35686003 PMC9171442

[45] Ghassemi Nejad J Vakili R Sobhani E Sangari M Mokhtarpour A Hosseini Ghafari SA. Worldwide research trends for chelates in animal science: A bibliometric analysis. Animals. (2023) 13:2374. doi: 10.3390/ani13142374 37508152 PMC10376876

[46] Khoshbin MR Vakili R Tahmasbi AM. Manganese-methionine chelate improves antioxidant activity, immune system and egg manganese enrichment in the aged laying hens. Vet Med Sci. (2023) 9:217–25. doi: 10.1002/vms3.1008 PMC9857133 36409287

[47] Reeves PG. Components of the AIN-93 diets as improvements in the AIN-76A diet. J Nutr. (1997) 127:838S–41S. doi: 10.1093/jn/127.5.838S 9164249

[48] National Research Council. Science and the Endangered Species Act. Washington (DC: National Research Council (1995).

